# Evaluation of Long-Term Outcomes of Direct Acting Antiviral Agents in Chronic Kidney Disease Subjects: A Single Center Cohort Study

**DOI:** 10.3390/jcm12103513

**Published:** 2023-05-17

**Authors:** Paulina Czarnecka, Kinga Czarnecka, Olga Tronina, Teresa Bączkowska, Aleksandra Wyczałkowska-Tomasik, Magdalena Durlik, Katarzyna Czerwinska

**Affiliations:** 1 Department of Transplantation Medicine, Nephrology and Internal Diseases, Medical University of Warsaw, 02-006 Warsaw, Poland; 2 Department of Immunology, Transplant Medicine and Internal Diseases, Medical University of Warsaw, 02-006 Warsaw, Poland

**Keywords:** hepatitis C infection (HCV), chronic infection, liver fibrosis, direct-acting antiviral agents (DAA), occult hepatitis C infection, treatment efficacy, hemodialysis, kidney transplantation

## Abstract

Background: The chronic kidney disease (CKD) population, including kidney transplant recipients (KTRs) and subjects on renal replacement therapy, is particularly vulnerable to unfavorable outcomes from chronic hepatitis C (CHC). Currently, there are oral direct-acting antiviral agents (DAAs) available to eradicate the virus with favorable short-term outcomes; however, their long-term effects are lacking. The aim of the study is to assess the long-term efficacy and safety of DAA therapy in the CKD population. Methods: An observational, cohort single-center study was performed. Fifty-nine CHC subjects with CKD, treated with DAAs between 2016 and 2018, were enrolled in the study. Safety and efficacy profiles were assessed, including sustained virologic response (SVR), occult hepatitis C infection (OCI) incidence, and liver fibrosis. Results: SVR was achieved in 96% of cases (n = 57). OCI was diagnosed only in one subject following SVR. Significant liver stiffness regression was observed 4 years after SVR compared to baseline values (Mdn = 6.1 kPa, IQR = 3.75 kPa; 4.9 kPa, IQR = 2.9 kPa), *p* < 0.001. The most common adverse events were anemia, weakness, and urinary tract infection. Conclusion: DAAs provide a safe and effective cure for CHC in both CKD patients and KTRs with a favorable safety profile in the long-term follow-up.

## 1. Introduction

Chronic hepatitis C (CHC) is a leading cause of liver-related diseases and mortality and a significant global health concern, affecting 71 million people worldwide [1]. CHC is far more prevalent in hemodialysis and kidney transplant recipients (KTRs) as compared to the general population [2]. In KTRs, the impact of CHC may even be more pronounced, given the permissive effect of immunosuppression on virus replication. 

Furthermore, the concomitance of chronic kidney disease (CKD) and CHC results in higher liver-related mortality, diminished quality of life, and greater cardiovascular risk and adverse graft outcomes [3,4,5]. CHC triggers accelerated deterioration of kidney function in subjects with pre-existing CKD and may cause CKD itself [6,7]. Currently, marketed antiviral therapies (AVTs) facilitate safe and effective hepatitis C virus (HCV) eradication in the CKD population [8,9]. In their most recent guidelines, the American Association for the Study of Liver Disease (AASLD) and the European Association for the Study of the Liver (EASL) recommend that all CKD subjects be considered for AVT [9,10]. Depending on the virus genotype and the advancement of both CKD and liver injury, CKD subjects may be offered grazoprevir in combination with elbasvir, glecaprevir with pibrentasvir, or sofosbuvir with velpatasvir. 

The previous CHC interferon (IFN)-based therapies were neither effective nor well tolerated and have been successfully displaced with oral direct antiviral agents (DAAs) [11]. The advent of DAAs has revolutionized HCV treatment, with an efficacy of 94–97% [12]. Similar efficacy has been firmly established with short-term observation of the CKD population [13,14]. Despite immense advancement in the CHC treatment landscape, long-term outcomes following DAA treatment completion in the CKD population are lacking.

Successful HCV eradication does not unequivocally indicate that the subject is completely cured. While viral clearance prevents further liver injury, histological and biochemical alteration in the liver may persist. Furthermore, several studies suggested that DAAs may not entirely eliminate the virus despite sustained virologic response (SVR), and viral genetic material may be identified in certain reservoirs. This phenomenon was termed occult HCV infection (OCI), a cell-specific type of HCV infection diagnosed by the detection of HCV RNA in liver tissue or peripheral blood mononuclear cells (PBMCs) despite consecutively negative detection of HCV RNA in serum with high-sensitive assays [15]. Two types of OCI are defined in the literature, including HCV seronegative or HCV seropositive subjects, in whom the virus has been eradicated spontaneously or with AVT [16]. This study will refer only to the latter type of OCI.

Depending on the detection method, HCV genetic material may be present in up to 83% of the cases upon viral clearance [17], which might be a reservoir for relapse and viral transmission, as suggested in some studies [18,19]. Others demonstrated that OCI might be a root cause of cryptogenic liver disease and abnormal biochemical liver function despite SVR [20,21]. Since current data on the importance of OCI remain inconclusive, our study aims to evaluate the implication and prevalence of OCI following viral clearance in CKD subjects. 

Undoubtedly, the primary goal of AVT therapy immediately after SVR is to prevent complications, which can be achieved by the prevention or regression of fibrosis. An increasing body of evidence revealed that a decline in liver stiffness (LS), to a certain extent, may be observed following successful HCV elimination [22]. Notably, liver fibrosis can be further utilized for the prognostication of adverse outcomes and patient mortality and may be perceived as an efficacy indicator of DAAs and a predictor of decompensation or hepatocellular carcinoma (HCC) occurrence in long-term follow-up [23,24]. 

Importantly, available studies have mostly documented a rapid decline in LS following SVR in a short time period; however, data beyond 12 months of observation are limited. It has been demonstrated that rapid fibrosis reversal measured with indirect fibrosis biomarkers might be overestimated, as it primarily stems from inflammation resolution, not the stiffness itself [25]. Therefore, it is important to verify the actual LS reduction that can be anticipated following AVT to accurately assess the long-term risk of CHC-related adverse outcomes. 

LS may be evaluated using both direct and indirect methods. While liver biopsy remains the gold standard for liver fibrosis assessment, this method can be neither widely applied in clinical practice nor recommended in the HCV population for surveillance purposes owing to its invasive nature. Therefore, non-invasive indicators of fibrosis are often employed, including the aspartate aminotransferase-to-platelet ratio (APRI), the Fibrosis Index Based on 4 Factors (FIB-4), and FibroScan transient elastography. These indirect biomarkers are validated fibrosis indicators in the HCV population, which allow repeated evaluation over time [26,27].

CHC has been recognized as a major cause of HCC [28]. HCC develops primarily in cirrhotic patients, and HCV-related cirrhosis entails a greater HCC risk as compared to cirrhosis of any other etiologies. As such, HCC surveillance is imposed in subjects with advanced fibrosis (F3–F4), even after HCV elimination [9]. Initial studies suggested an association between DAAs and HCC development, which was not subsequently confirmed by several large-cohort studies [29,30,31]. Nevertheless, there is little doubt that the residual HCC risk persists after HCV eradication [32,33].

Until recently, CKD subjects were often left untreated despite the excellent efficacy of DAAs, as it was unclear whether new antiviral agents exacerbate kidney deficiency. Most recent studies showed that DAAs did not significantly impact kidney function or might even alleviate the decrease in estimated glomerular filtration rate (eGFR) in the CKD population upon SVR [34,35]. However, investigations of long-term kidney outcomes following SVR are limited. 

Evidence suggests that DAA treatment may trigger hepatitis B (HBV) reactivation even after AVT completion exists; therefore, HBV status needs to be established prior to DAA commencement [9,36,37]. Current HBV infection may require HBV nucleoside/nucleotide analog administration while receiving DAAs, whereas a history of HBV infection mandates close monitoring of alanine aminotransferase (ALT). Whenever ALT elevation persists after the end of treatment (EOT) or occurs during AVT, the subject needs to be tested for HBsAg and HBV DNA. Of note, patients on renal replacement therapy and KTRs are particularly vulnerable to HBV infection since HBV infection is far more prevalent in these subjects compared to the general population and often does not entail ALT elevation. Data on HBV reactivation in these populations are lacking. 

To our best knowledge, this is the first study to report long-term data on fibrosis and kidney function assessment with a focus on the CKD population who receive IFN-sparing regimens. We consider this study of great importance as the CKD population is particularly vulnerable to unfavorable liver outcomes from CHC.

## 2. Materials and Methods

### 2.1. Study Population and Design

**Scheme 1:**
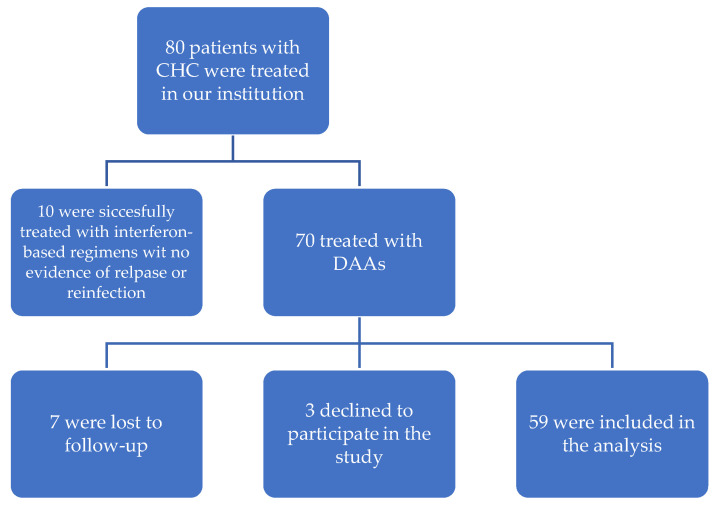
Study population.

In this observational, single-center cohort study, all subjects with CHC and CKD (hemodialysis-dependent, KTRs, and ESRD), who underwent DAA therapy at our institution between 2016 and 2018 with valid FibroScan evaluation prior to DAA treatment and were able to provide written informed consent, were eligible for inclusion (Scheme 1). Subjects with coexisting HIV infection were excluded. Both treatment-naive and treatment-experienced patients were of interest. CHC was confirmed using HCV RNA polymerase chain reaction (PCR) detection (Cobas^®^ AmpliScreen HCV v1.0 with the lower limit of detection of 15 IU/mL; Roche Diagnostics, Branchburg, NJ, USA) for at least 6 months. HCV genotyping was performed using the Linear Array Genotyping Test and Cobas^®^ TaqMan^®^ Qualitative test v1.0 with the lower limit of detection of 21 UI/mL (TaqMan; Roche Diagnostics, Branchburg, NJ, USA). 

Data from medical records were obtained for concentrations of total bilirubin, aspartate aminotransferase (AST), ALT, and gamma-glutamyl transpeptidase (GGT) activity, albumin, and complete blood count, which were measured during routine outpatient visits before treatment, at EOT, and 1, 2, and 4 years after EOT. Similarly, indirect fibrosis biomarkers were evaluated, whereas FibroScan was conducted prior to AVT and at 4 years after EOT. Women of childbearing age were monitored with serum B-HCG while receiving DAAs. Kidney function was assessed by serum creatinine concentration (Scr) and eGFR estimated per the CKD Epidemiology Collaboration (CKD-EPI) equation. An allograft biopsy in KTRs was obtained whenever clinically indicated by the attending physician and assessed by the Banff criteria [38]. Data on AEs, CNI dose adjustments, and allograft biopsy reports were retrieved from medical records.

All subjects were screened for HCC with alfa-fetoprotein (AFP) and ultrasound examination prior to DAA commencement and remained under surveillance for HCC during the observation period. HBV status (HBsAg and anti-HBc) was established prior to DAA commencement, and anti-HBc-positive subjects were monitored monthly for HBV reactivation with ALT, HBsAg, and HBV DNA. 

All SVR subjects had blood samples collected for OCI and underwent FibroScan transient elastography at 4 years after EOT. HCV RNA in PBMCs was tested in two consecutive samples, which were collected in intervals of 2–3 months. 

AVT was based on DAAs with or without RBV. DAA therapy and its duration were guided by virus genotype, viral load (expressed as log_10_Iu/mL), kidney function, previous treatment status, liver disease severity, and DAA availability [39,40]. Detailed information on the use of AVT is available in Table 1. 

The efficacy of DAA treatment was defined as SVR, which was determined by undetectable HCV RNA by PCR at 12 weeks after therapy completion. Patients who achieved SVR were re-tested for HCV RNA at the time of blood collection for PBMCs to discriminate between OCI and HCV reinfection.

All subjects provided informed consent form prior to study enrollment. The study was conducted in accordance with the provisions of the Declaration of Helsinki, and a favorable opinion of the Ethics Committee of the Medical University of Warsaw was obtained (KB/159/2019).

### 2.2. PBMC Isolation from Whole Blood

About 10 mL of whole blood was collected in a sterile EDTA-containing tube and diluted with NaCl (1:1). Immediately after collection, PBMCs were separated from whole blood with density gradient centrifugation and Ficoll Hypaque (Lonza, Verviers, Belgium) per manufacturer’s instructions. Afterward, cells were washed three times with phosphate-buffered saline (PBS, pH 7.3 ± 0.1). The PBMCs were resuspended in RNALater solution (Ambion Inc., Austin, TX, USA) and stored at −80 °C for further analysis.

### 2.3. Detection of HCV RNA in PBMCs 

HCV RNA was analyzed in PBMC with real-time PCR (RT-PCR) using COBAS AmpliPrep/COBAS TaqMan HCV Quantitative Test v2.0 (Roche Diagnostics, Basel, Switzerland) and COBAS TaqMan Analyzer COBAS AmpliPrep Instrument analyzer (Roche Diagnostics, Basel, Switzerland) according to three control levels: Roche Diagnostics COBAS TaqMan Negative Control, HCV Low Positive Control, and HCV High Positive Control. The assay was performed in a PBMCs sample volume of 500 µL per the manufacturer’s recommendation. PBMC pellets were analyzed upon 1-min centrifugation at 3000 relative centrifugal force (rcf) with the Eppendorf Centrifuge 5424 (Eppendorf, Hamburg, Germany). HCV RNA concentration was automatically calculated using AMPLILINK 3.3.7 software (Roche Diagnostics, Basel, Switzerland).

### 2.4. Liver Fibrosis Assessment

LS was assessed with non-invasive biomarkers, such as APRI and FIB-4 scores, and transient elastography (FibroScan^®^ Mini +430, Echosens, Paris, France). FibroScan was performed by a certified physician and graded per the Metavir scale as follows: F0–F1 (≤7 kPa), F2 (7.1–9.4 kPa), F3 (9.5–12.4 kPa), and F4 (≥12.5 kPa) [41]. Only FibroScan reports with at least 10 valid measurements, a success rate of at least 60%, and an interquartile range (IQR) < 0.3 were considered valid. LS was reported in the unit of kPa. Indirect fibrosis biomarkers, APRI [42] and FIB-4 [43], were computed based on the following equations:

APRI = (AST [IU/L]/upper limit of the normal AST range*)/platelet count [10^9^/L] × 100

* the upper limit of normal AST range was 50 IU/L.

FIB-4 = (age [years] × AST [IU/L]/platelet count [10^9^/L] × √ALT [U/L])

### 2.5. Statistical Analysis

Analyses were conducted using the R Statistical language (version 4.1.1) on Windows 10 x64 (build 19044), using the packages effect size (version 0.6.0.1), sjPlot (version 2.8.9), report (version 0.5.1), ggstatsplot (version 0.9.0), and psych (version 2.1.6). The significance level of statistical tests in this analysis was set at α = 0.05.

The normality of the variables was tested using the Shapiro–Wilk test. Additionally, measurements of asymmetry (skewness) and shape (kurtosis) were taken into account. Distributions in which skewness did not exceed 2.0 and kurtosis was below 7.0 were considered normal.

For non-parametric analyses of more than two groups, the Friedman rank sum test with Kendall’s coefficient of concordance was used. For parametric analyses of more than two groups, Fisher’s repeated measures one-way analysis of variance (ANOVA) with omega-squared effect size was conducted.

For analyses of two groups with a normally distributed variable, Student’s *t*-test was used with the effect size calculated as Hedges’ g. For variables without a normal distribution, the Wilcoxon test was applied with the estimation of a biserial rank correlation.

The correlation test statistics were based on Pearson’s product-moment correlation coefficient and followed a *t* distribution with the length (x)–−2 degrees of freedom. An asymptotic confidence interval (CI) was given based on Fisher’s *Z* transform.

The relationship between the nominal value and a continuous value was estimated with a point biserial correlation coefficient. Regression analysis in terms of cardinal and interval data was based on the linear model (GLMM).

### 2.6. Aim and Study Endpoints

This analysis aimed to assess the long-term efficacy and safety of DAAs in the CKD and end-stage renal disease (ESRD) population.

The primary study endpoints were SVR at 12 weeks following AVT cessation and OCI incidence following SVR, liver fibrosis estimated using FibroScan 4 years after SVR.

The secondary outcomes included renal function evaluated with Scr and eGFR, liver fibrosis estimated by indirect fibrosis biomarkers (APRI and FIB-4 scores), treatment-related adverse events (AEs), HCC incidence over a 4-year follow-up, HBV reactivation, liver function estimated using ALT, AST, and GGT, and the number of calcineurin inhibitor (CNI) dose adjustments.

## 3. Results

A total of 59 patients were enrolled (treatment-naive: 79.7%) with a mean age of 48.8 years (18–70 years) and a male ratio of 62.7% (n = 37). The study population consisted of 51 KTRs (all from deceased donors), 7 hemodialysis-dependent subjects, and 1 ESRD subject. The predominant HCV genotype was 1b (64.4%), followed by genotype 4 (23.7%). The median viral load was 1.97 × 10^6^ (IQR: 3.22 × 10^6^). The vast majority (64%) of the subjects had F0–F1 fibrosis at baseline. The leading cause of ESRD was glomerulonephritis (40.7%). The mean time from kidney transplantation (KTx) to AVT commencement for KTRs was 11.18 years (standard deviation [SD]: 7.49 years). Most KTRs were maintained on a triple immunosuppressive scheme consisting of tacrolimus, mycophenolate mofetil, and prednisone. A history of HBV infection (hBsAg-, anti-HBc+) was present in 39% of the subjects. Patients were treated with IFN-sparing regimens (sofosbuvir-based: 52.6%, combined with rybavirin (RBV): 76.2%). AVT was administered for 8–24 weeks. Detailed baseline group characteristics are presented in Table 1.

### 3.1. SVR

SVR was achieved in 57/59 patients (96.6%). One patient without viral clearance (virus genotype 4) was on renal replacement therapy and was treatment-experienced (previously received pegylated IFN and simeprevir combined with RBV). The other one was a treatment-naive KTR with virus genotype 3. Both were men with baseline cirrhosis (F4) and suspected non-compliance (subjects did not recall the AVT schedule, and tablet counts significantly deviated from the prescribed frequency during follow-up visits). Excessive alcohol intake was confirmed later for both individuals. Following DAA failure, blood samples were collected and sent for analysis to identify any potential underlying resistant mutations that could have impacted unfavorable DAA outcomes. Results for both subjects were unremarkable for the resistance-associated NS5A mutations. No non-responders were retreated with DAAs due to active alcoholism. The first one died due to HCC and rapid progression of liver decompensation two years after AVT. The other subject died from a cardiovascular event one year after AVT.

### 3.2. Kidney Function

**Figure 1:**
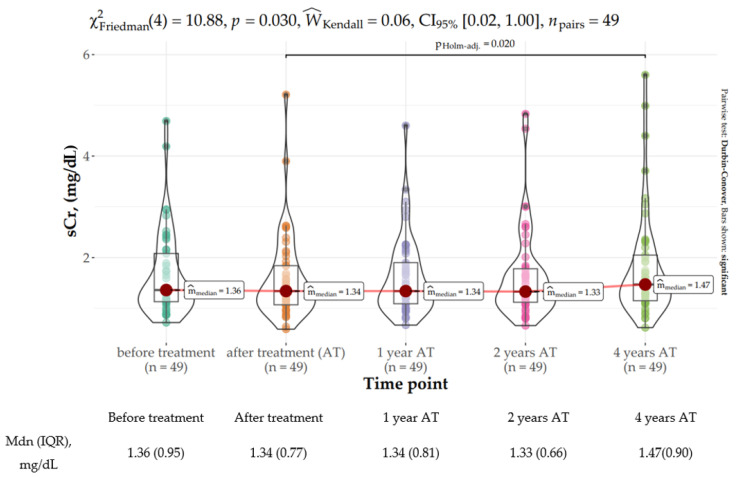
Distribution of serum creatinine concertation (mg/dL) at baseline, EOT (end of treatment), and 1, 2, and 4 years following sustained virologic response (SVR).

Scr remained stable for the duration of AVT and until 2 years after SVR. Overall, there was a significant increase in Scr 4 years after EOT (median: 1.47 mg/dL, IQR: 0.9 mg/dL) as compared to the baseline value (median: 1.34 mg/dL, IQR: 0.77 mg/dL) (χ^2^_Friedman_ (4) = 10.88, *p* = 0.028, Figure 1). The effect size was slight per Landis’s (1997) conventions. Kidney function deterioration was noted in 42.9% of the subjects (n = 21), whereas Scr improved at the 4-year observation timepoint in 32.6% of the subjects (n = 16) and remained intact in another 24.5% of the subjects (n = 12). Further details are provided in Figure 1.

**Figure 2:**
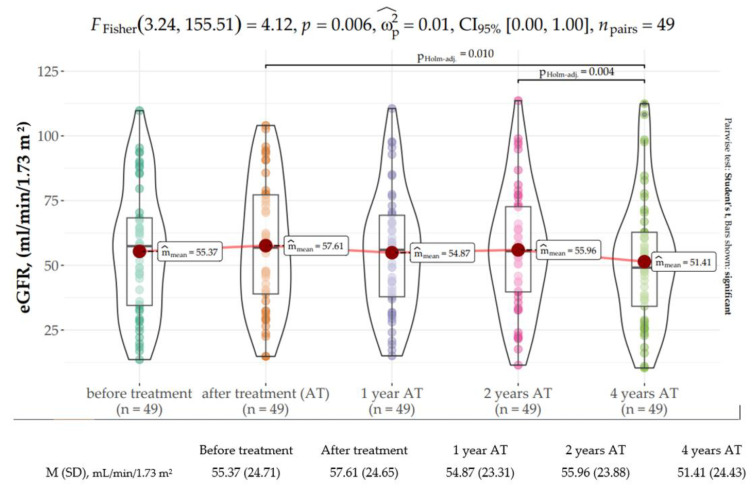
Distribution of the estimated glomerular filtration rate (eGFR, mL/min/1.73 m^2^) at baseline, end of treatment (EOT), and 1, 2, and 4 years following sustained virologic response (SVR).

Similarly, kidney function delineated by the mean eGFR was comparable while on AVT. Nevertheless, Fisher’s repeated measures one-way ANOVA revealed that eGFR gradually declined over time upon SVR, reaching a statistically significant decrease at 4 years after SVR (mean: 57.61 mL/min/1.73 m^2^, SD: 25.41 mL/min/1.73 m^2^ vs. mean: 51.41 mL/min/1.73 m^2^, SD: 24.43 mL/min/1.73 m^2^) (F_Fisher_ [3.24, 155.51] = 4.12, *p* = 0.006, Figure 2). The effect size was very small per Field’s (2013) conventions. The most prominent decline of 4.55 mL/min/1.73 m^2^ was observed between the second and fourth years of observation.

### 3.3. Liver Function

ALT sharply declined at EOT as compared to the baseline value (median: 20 U/I, IQR: 7.5 U/I vs. median: 40 U/I, IQR: 42.5 U/I; *p* < 0.001) and further decreased until one year after EOT (median: 14 U/I, IQR: 9 U/I; *p* < 0.001). After one year, ALT plateaued within the range of normality (see Table 2). Consistently, AST and GGT markedly declined after treatment and remained within the range of normality thereafter (see Table 2).

**Figure 3:**
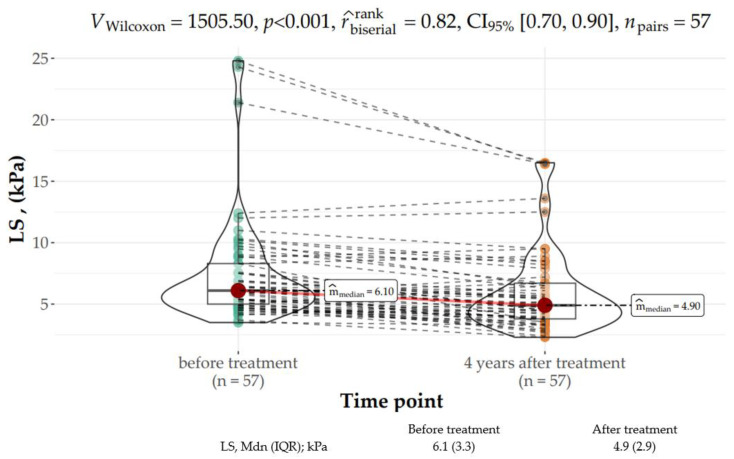
Distribution of LS (kPa) parameters at baseline and 4 years after the end of treatment (EOT).

The V Wilcoxon test for dependent variables revealed that LS significantly improved 4 years following SVR as compared to the baseline value (median: 6.1 kPa, IQR: 3.75 kPa vs. median: 4.9 kPa, IQR: 2.9 kPa) (V Wilcoxon 1500, *p* < 0.001 with strong correlation coefficient, Figure 3). Two subjects who did not achieve SVR were not available for LS assessment at the 4-year time point.

**Figure 4:**
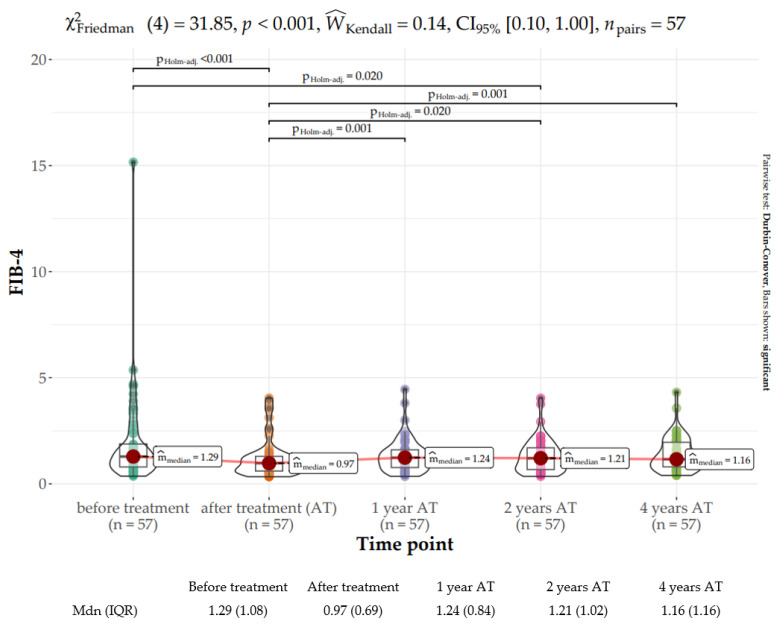
Distribution of the Fibrosis Index Based on 4 Factors (FIB-4) score at baseline, end of treatment (EOT), and 1, 2, and 4 years following sustained virologic response (SVR).

The Friedman rank sum test showed a statistically significant difference between the baseline FIB-4 score (median: 1.29, IQR: 1.3) with the EOT score (median: 0.97, IQR: 0.82) as well as the score at two years after treatment (median: 1.21, IQR: 1.07) (χ^2^_Friedman_ (4) = 31.85, *p* < 0.001). The effect size was slight per Landis’s (1997) conventions (Figure 4).

The FIB-4 score at EOT was significantly lower than those at later time points. The scores of the remaining time points after treatment did not differ significantly from each other and were in the range of 1.16–1.24.

**Figure 5:**
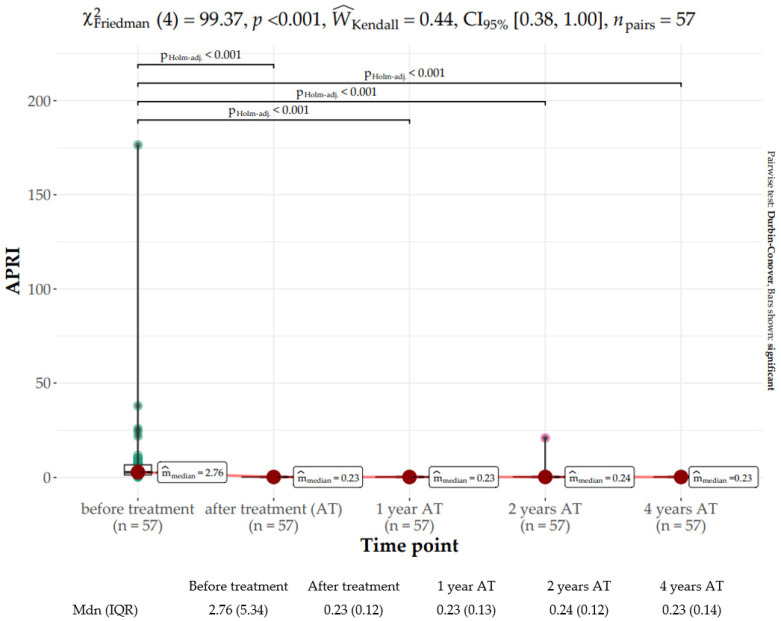
Distribution of the aspartate aminotransferase-to-platelet ratio (APRI) score at baseline, end of treatment (EOT), and 1, 2, and 4 years following sustained virologic response (SVR).

The APRI score notably improved from baseline (median: 2.76, IQR: 5.36) to EOT (median: 0.23, IQR: 0.12) and plateaued within the range of 0.23–0.24 thereafter (χ^2^_Friedman_ (4) = 99.37, *p* < 0.001). The effect size was moderate per Landis’s (1997) conventions (Figure 5).

Both APRI (r = 0.53, *p* < 0.001) and FIB-4 (r = 0.70; *p* < 0.001) scores were strongly correlated with fibrosis measured with FibroScan transient elastography before AVT initiation. However, at the 4-year observation time point, only APRI (r = 0.33, *p* = 0.013) score, but not the FIB-4 score (r = 0.25, *p* = 0.063), showed a moderate correlation.

**Figure 6:**
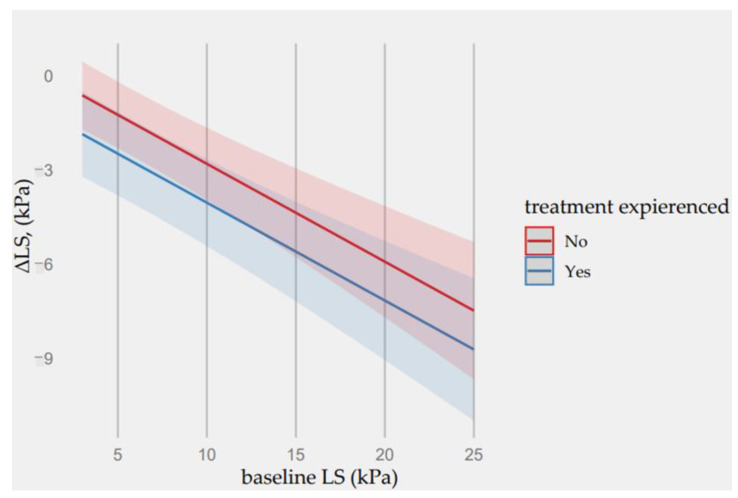
Predictions of ΔE values from the fitted regression model based on baseline LS concentration and patient’s treatment-experienced factor.

Neither genotype nor liver fibrosis at baseline impacted liver fibrosis stiffness regression. Only in treatment-experienced patients, a greater liver fibrosis reversal of 1.24 (CI: −2.27 to −0.21, *p* = 0.020) was observed. Further details are provided in Figure 6.

**Figure 7:**
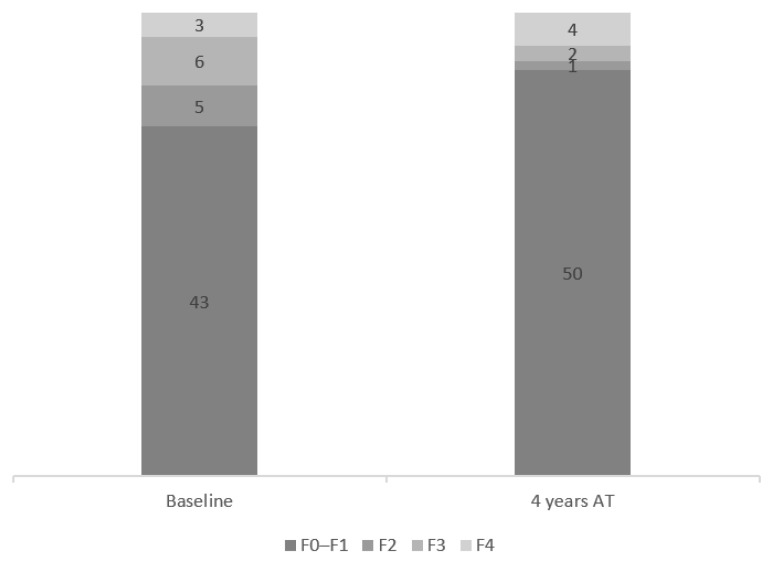
Fibrosis distribution at baseline and at 4 years after end of treatment (EOT).

At the 4-year time point, the percentage of subjects with advanced fibrosis (F3–F4) estimated per FibroScan was reduced from 19.83% (n = 11) to 12.3% (n = 7). While fibrosis was not downgraded in any cirrhosis patients, this parameter was downgraded to F2 or even F1 at the end of the follow-up in four patients with advanced fibrosis (F3). In one subject with advanced fibrosis, this condition had progressed to cirrhosis based on FibroScan measurements (baseline vs. 4 years after EOT: 12.4 kPa vs. 13.6 kPa). Further details are provided in Figure 7.

### 3.4. CNI Dose Adjustments

CNI was administered to 81.4% of the study subjects. All subjects had their CNI dose reduced prior to AVT commencement whenever ritonavir was used. No immunosuppression dose adjustments were required for 24.5% of the patients, whereas more than 20% of the patients required more than 2 CNI dose adjustments during DAA therapy. 

### 3.5. Safety

Overall, 102 AEs were observed, with the majority being mild to moderate in intensity. The most commonly reported AEs were anemia (25.4%), weakness (22%), and urinary tract infection (UTI, 22%). UTI was not considered AVT-related. Anemia was observed only in RBV-treated subjects. Twenty-one subjects (19.1%) did not experience any AEs. Serious AEs (SAEs) requiring hospitalization were reported for eight patients. The vast majority (n = 5) was due to UTI, followed by pneumonia and atrial fibrillation. No SAEs were considered AVT-related or resulted in treatment discontinuation. Detailed information on AEs is present in Table 3. 

During the study, renal biopsy was clinically indicated in one subject while on DAAs. The biopsy report was indicative of acute antibody-mediated rejection (ABMR). This 32-year-old male patient, for whom DAA was initiated at 6 months after KTx, had biopsy-proven ABMR prior to HCV treatment. As such, AMBR was not considered treatment-induced.

No HBV reactivation was noted during the observation period.

HCC was observed in two subjects, including one who did not achieve SVR. The subject, who was successfully treated for CHC, had genotype 3; and a single HCC lesion was diagnosed in segment VII 6 months after EOT despite no evidence of HCC prior to DAA initiation. He had F3 fibrosis at baseline, and no liver fibrosis regression was observed with both serum fibrosis biomarkers and liver elastography. He was successfully treated for HCC with tumorectomy and remained in the follow-up with no evidence of HCC recurrence.

### 3.6. OCI

HCV was detected in PBMCs only in one subject and in both samples. This patient was a treatment-experienced 28-year-old man after 3 KTx with virus genotype 1b, who was on a triple immunosuppressive scheme with sofosbuvir-based regimens and RBV. He did not have any evidence of liver injury, his ALT was within the normal range, and HCV RNA was undetectable in serum 4 years following EOT. Regardless of OCI, fibrosis regression was observed in this patient from 5.4 kPa to 2.8 kPa, and there was no evidence of HCC during the observation period.

### 3.7. Long-Term Outcomes of DAAs in Kidney Transplant Recipients

The Friedman rank sum test revealed that the FIB-4 score significantly declined between baseline (median = 1.38, IQR = 1.42) and EOT (median = 1.10, IQR = 0.81) and then gradually increased over time, reaching median = 1.25, IQR = 1.16 four years after EOT (χ^2^_Friedman_ (4) = 31.85, *p* < 0.001). The effect size was slight, as per Landis’s (1997) conventions (see Figure S1).

Similarly, a notable change in APRI score was noted between baseline (median = 3.12, IQR = 5.57) and EOT (median = 0.24, IQR = 0.12) χ^2^_Friedman_ (4) = 86.45, *p* < 0.001, and plateaued within the rage of 0.23–0.24. The effect size was moderate, as per Landis’s (1997) conventions (see Figure S2).

The *V* Wilcoxon test for dependent variables revealed a significant difference in *LS* before treatment (median = 6.05 kPa, IQR = 3.75 kPa) and four years after treatment (median = 4.80 kPa, IQR = 277 kPa), *V_Wilcoxon_*= 1200.50, *p* <0.001 with strong correlation coefficient (see Figure S3). 

ALT rapidly declined at EOT when compared to the baseline value (median = 40.50 U/I, IQR = 47.75 U/I vs. median = 20.50 U/I, IQR = 7.75 U/I; *p* < 0.001) and further decreased until one year after EOT (median = 15.00, IQR = 8.75; *p* < 0.001). After one year, ALT plateaued within the range of normality (Figure S4). Consistently, AST and GGT markedly declined after treatment and remained within the range of normality thereafter (Figures S5 and S6).

## 4. Discussion

The present study demonstrated that DAAs were highly effective and well-tolerated in both ESRD patients and KTRs, with the SVR rate exceeding 96%. This finding is congruent with those from previously published studies in CKD cohorts [13,14]. 

Further, OCI infection was infrequent in the CKD population following SVR, and no adverse clinical implications of HCV RNA in PBMCs were detected. Despite having detectable HCV RNA in PBMCs, the only OCI-positive subject had fibrosis reversal by 48% at the 4-year follow-up time point with no evidence of viral relapse. Lybeck et al. demonstrated a similarly low OCI incidence during their long-term follow-up study [44]. However, they did not observe fibrosis alleviation when OCI was diagnosed. Since the OCI subject in our analysis had milder fibrosis as estimated with FibroScan, we speculated that his condition was more likely reversible. Furthermore, unlike Lybeck et al., who examined the long-term impact of IFN-based agents, we analyzed the impact of DAAs on fibrosis; there is current evidence suggesting that greater fibrosis regression may be anticipated after DAAs as compared to IFN-based therapies [22,45]. Despite the advocacy against the clinical implication of OCI in our study, its results need to be interpreted with caution, given the small sample size, and further studies are needed to elucidate these findings.

Further, the OCI incidence in our study may be underestimated, as PBMCs might reveal only up to 61% of the OCI cases as compared to liver biopsy [46]. Given the invasive nature of the biopsy with limited benefit for the subject, we decided against this method of OCI detection for our study purpose. In the literature, OCI incidence following SVR varied, with ranges of 0–50% in PBMCs and 0–83% in liver tissue [17]. Discrepancies in OCI incidence may stem from differences in detection methods among the studies. A uniform approach for OCI detection would be important to determine the true incidence rate and implication following SVR.

The present study demonstrated the long-term effectiveness of DAA. Fibrosis decline was documented with both FibroScan transient elastography and indirect serum biomarkers. Four years after AVT cessation, fibrosis regression by 20%, as estimated with FibroScan, was observed. However, SVR could not be identified with complete cure as advanced fibrosis or cirrhosis was still present in as much as 12% of the subjects after EOT. In other studies, higher LS decline was observed in the general population [22,33,45]. Facciorusso et al. documented an LS decline of 46% at 5 years in 83 DAA responders, whereas Flisiak et al. documented an LS decline exceeding 30% at 5 years [33,45]. Importantly, their data referred to the general population and could not be easily applied to the CKD population. Additionally, the majority of the population in our study comprised KTRs, with CNI, MMF, and GKS being the most prevalent immunosuppressive regimens. Since CNIs and MMF are known to exert profibrotic properties, we may speculate that immunosuppressive treatment could have contributed to the lower regression of LS [47,48,49]. Additional studies are needed to prognosticate the extent to which fibrosis regression could be anticipated after SVR in CKD subjects.

Interestingly, the only predictor of greater LS decline was treatment-experienced status. However, among the group with available paired LS measurements, fibrosis was lower at baseline, and two subjects from this group did not reach SVR. Therefore, this finding needs to be interpreted with caution. Previous studies also aimed to identify potential predictors of fibrosis decline. Both host factors, such as the presence of liver cirrhosis, old age of the patient, alcohol consumption, diabetes mellitus or BMI, and viral factors (genotype, viral load) were investigated, but the results remained inconclusive [50,51]. 

Even though non-invasive fibrosis indices decreased significantly after DAA therapy, they only showed a moderate correlation with FibroScan results, which might translate to lesser reliability for follow-up purposes after SVR. Przekop et al. observed that the correlation between fibrosis estimated with indirect serum biomarkers and FibroScan decreased after treatment [52]. Similar observations have been previously noted in HBV subjects [53]. Since both FIB-4 and APRI scores include the ALT value in their formulas, these scores may automatically decline with the reduction in inflammation despite the persistence of fibrosis, which may explain the weaker correlation with FibroScan following SVR.

In our analysis, both Scr and eGFR remained intact at EOT. Moreover, no acute kidney injury (AKI) incidence was observed during AVT. Since on-treatment raw creatinine values were not collected for the purpose of this analysis, we could not exclude eGFR fluctuations. This finding is supported by a recent analysis by Sulkowski et al., who demonstrated that even sofosbuvir-based AVT, which was considered nephrotoxic until recently, did not have an adverse impact on kidney function [54].

Furthermore, in our study, one year after EOT, kidney function progressively deteriorated, reaching a statistically significant decline at the 4-year observation time point. This progressive decline in kidney function was similarly reported in other studies, such as Saxena et al. [55]. While available analysis showed relatively stable kidney function in terms of AVT, most studies were of limited follow-up duration, and the long-term effects of AVT have not been fully elucidated [56,57,58,59].

In our study, the most prominent decline in eGFR was observed between the 2- and 4-year observation time points. Importantly, the vast majority of our patients comprised KTRs, with an average duration of 11 years from KTx until DAA commencement. Since it has been previously documented that kidneys from deceased donors last an average of 12 years, we may presume that the observed kidney function deterioration was rather a result of kidney allograft lifespan triggered by AVT [60]. Nevertheless, it is not surprising that kidney biomarkers may deteriorate during follow-up due to the progressive nature of CKD. Furthermore, a retrospective observational cohort study by Sise et al. demonstrated that AVT might not prevent eGFR decline entirely in the CKD population but only slow down CKD progression [34]. Nevertheless, it has been firmly established that SVR conferred a survival benefit in dialysis patients and reduced ESRD risk, which are of greater importance than eGFR. Additional studies are needed to evaluate eGFR dynamics following DAA beyond one year of observation.

DAAs were well-tolerated, and only a limited number of AEs were reported during AVT. Acute rejection was also infrequent and considered not AVT-related. The most commonly reported AEs were RBV-induced anemia, weakness, and UTI. Nevertheless, no red blood cell transfusion was required, and RBV dose reduction or discontinuation was needed in all subjects receiving RBV. Likewise, RBV reduction was clinically indicated in other studies [61]. The vast majority of our study population comprised KTRs, and it has been previously documented that infections are the most prevalent complication following KTx, with UTI being the most frequent. No UTIs were AVT-related. Furthermore, interactions with CNIs could be limited under the current CHC treatment landscape with the use of pan-genetic DAA regimens. Since RBV is rarely added to the AVT scheme, we might assume that AEs might even be less frequent [8].

Genotype 3 is considered more difficult to treat when compared to the remaining genotypes, as it entails a greater risk of virologic failure [39]. In our study, 4 out of 5 patients with genotype 3 achieved SVR. The one who did not was suspected of non-compliance, as described above. 

The present study has some limitations. First, the study was of a retrospective nature with a small sample size. Additionally, prescribed CHC agents at the time when the study was conducted are not in line with the most recent recommendations. Moreover, there is a lack of consistency among CHC therapies used and histological validation of fibrosis assessments, which were measured with indirect fibrosis indices. However, the completeness of data and long-term follow-up period are some definite strengths of our study.

Despite the great achievement in the field of HCV eradication, there are still many challenges ahead, including the goal of HCV elimination by 2030, variations in medication availability among different countries, and high treatment costs. 

## 5. Conclusions

DAAs provided a safe and effective cure for CHC in both CKD patients and KTRs with a favorable safety profile. Long-term liver fibrosis regression might be obtained with AVT. There was no evidence of OCI in CKD subjects and KTRs following SVR. Longitudinal studies are needed to establish whether post-SVR liver regression translates to mortality and morbidity benefit.

## Figures and Tables

**Table 1:** Baseline group characteristics.

Variable		N = 59
Sex		
Male	N (%)	37 (62.7)
Female	N (%)	22 (37.3)
Age, years	Mean (SD)	48.8 (13.1)
Concomitant disease		
Hypertension	N (%)	51 (86.4)
Diabetes	N (%)	21 (35.6)
Dyslipidemia	N (%)	32 (54.2)
Number of Ktx		
1	N (%)	26 (44.1)
2	N (%)	18 (30.5)
3	N (%)	7 (11.9)
Genotype		
1a	N (%)	2 (3.4)
1b	N (%)	38 (64.4)
3	N (%)	5 (8.5)
4	N (%)	14 (23.7)
HCV viral load	Median (IQR)	1.97 × 10^6^ (3.22 × 10^6^)
Treatment status		
Experienced	N (%)	12 (20.3)
Naive	N (%)	47 (79.7)
DAA therapy used		
Ombitasvir/parytaprevir/rytonavir	N (%)	14 (23.7)
Ledipasvir/sofosbuvir	N (%)	31 (52.5)
Glecaprevir/pibrentasvir	N (%)	3 (5.1)
Elbasvir/grazoprevir	N (%)	11 (18.6)
RBV	N (%)	45 (76.2)
Treatment duration		
8 weeks	N (%)	2 (3.4)
12 weeks	N (%)	49 (83.0)
16 weeks	N (%)	2 (3.4)
24 weeks	N (%)	6 (10.2)
Immunosuppression		
CNI	N (%)	48 (81.4)
MMF	N (%)	45 (76.3)
GKS	N (%)	53 (89.9)
Time since Ktx and DAAs, years	Mean (SD)	11.18 (7.49)
BMI, kg/m^2^	Mean (SD)	24.2 (4.3)
Past HBV infection		
Yes	N (%)	23 (39.0)
No	N (%)	36 (61.0)
ESRD		
Glomerulonephritis	N (%)	24 (40.7)
Unknown etiology	N (%)	7 (11.9)
Hypertension	N (%)	3 (5.1)
Diabetes	N (%)	8 (13.6)
Vasculitis	N (%)	1 (1.7)
Congenital urinary tract defect	N (%)	6 (10.2)
Obstructive nephropathy	N (%)	4 (6.8)
Chronic interstitial nephritis	N (%)	4 (6.8)
HUS	N (%)	2 (3.4)
Fibrosis		
LS, kPa	Median (IQR)	6.1 (3.75)
F0–F1	N (%)	38 (64.4)
F2	N (%)	10 (16.9)
F3	N (%)	6 (10.2)
F4	N (%)	5 (8.5)
FIB-4	Median (IQR)	1.33 (1.3)
APRI	Median (IQR)	2.76 (5.36)
CAP, dB/m	Mean (SD)	231.19 (44.0)

APRI, aspartate aminotransferase-to-platelet ratio; BMI, body mass index; LS, liver stiffness; CAP, Controlled Attenuation Parameter; CNI, calcineurin inhibitors; DAA, direct oral antiviral agents; ESRD, end-stage renal disease; FIB-4, Fibrosis Index Based on 4 Factors; GKS, glucocorticosteroids; HBV, hepatitis B virus; HCV, hepatitis C virus; HUS, hemolytic uremic syndrome; IQR, interquartile range; Ktx, kidney transplantation; MMF, mycophenolate mofetil; RBV, ribavirin; SD, standard deviation.

**Table 2:** Distribution of liver function tests at baseline, end of treatment (EOT), and 1, 2, and 4 years following sustained virologic response (SVR).

Variable	Before Treatment	After Treatment	1 Year AT	2 Years AT	4 Years AT
ALT Mdn (IQR), U/I	40.0 (40.0)	20.0 (8.0)	14.0 (9.0)	15.0 (7.0)	15 (9.0)
AST Mdn (IQR), U/I	33.0 (24.0)	19.0 (11.0)	20.0 (7.0)	19.0 (6.0)	18.0 (8.0)
GGT Mdn (IQR), U/I	61.0 (72.0)	27.0 (27.0)	24.0 (22.0)	28.0 (23.0)	26.0 (18.0)

**Table 3:** Adverse events.

Adverse Event		N = 59
Anemia	N (%)	15 (25.4%)
Weakness	N (%)	13 (22%)
Urinary tract infection	N (%)	13 (22%)
Sterile leukocyturia	N (%)	8 (13.6%)
Upper respiratory tract infection	N (%)	7 (11.9%)
Headache	N (%)	4 (6.8%)
Diarrhea	N (%)	3 (5.1%)
Hypertransaminasemia	N (%)	3 (5.1%)
Pneumonia	N (%)	3 (5.1%)
Abdominal pain	N (%)	2 (3.4%)
Ankle edema	N (%)	2 (3.4%)
Worsening of blood pressure control	N (%)	2 (3.4%)
Herpes	N (%)	2 (3.4%)
Fatigue	N (%)	2 (3.4%)
Worsening of exercise tolerance	N (%)	1 (1.7%)
Bone pains	N (%)	1 (1.7%)
Distal deep vein thrombosis	N (%)	1 (1.7%)
Nausea	N (%)	1 (1.7%)
Sleep disturbance	N (%)	1 (1.7%)
Hematuria	N (%)	1 (1.7%)
Weight loss	N (%)	1 (1.7%)
Syncope	N (%)	1 (1.7%)
Hyperkalemia	N (%)	1 (1.7%)
Gout	N (%)	1 (1.7%)
Sinusitis	N (%)	1 (1.7%)
Hypotension	N (%)	1 (1.7%)
Fistulae	N (%)	1 (1.7%)
Atrial fibrillation	N (%)	1 (1.7%)
Inflammatory fistula tumor	N (%)	1 (1.7%)
Otitis	N (%)	1 (1.7%)
Pruritus	N (%)	1 (1.7%)

## Data Availability

Data supporting the results reported in the article can be found under the following DOI: 10.17632/8yrhk6gv8n.1.

## Supplementary Materials

The following supporting information can be downloaded at: https://www.mdpi.com/article/10.3390/jcm12103513/s1. Figure S1: Distribution of the FIB-4 parameter at baseline, end of treatment (EOT), and 1, 2, and 4 years following sustained virologic response (SVR) in kidney transplant recipients; Figure S2: Distribution of APRI parameter at baseline, end of treatment (EOT), and 1, 2, and 4 years following sustained virologic response (SVR) in kidney transplant recipients; Figure S3: Distribution of LS (kPa) parameters at baseline and 4 years after the end of treatment (EOT) in kidney transplant recipients; Figure S4: Distribution of alanine aminotransferase (ALT, U/L) at baseline, end of treatment (EOT), and 1, 2, and 4 years following sustained virologic response (SVR) in kidney transplant recipients.; Figure S5: Distribution of aspartate aminotransferase (AST, U/L) at baseline, end of treatment (EOT), and 1, 2, and 4 years following sustained virologic response (SVR) in kidney transplant recipients.; Figure S6: Distribution of gamma-glutamyl transferase (GGT, U/L) at baseline, end of treatment (EOT), and 1, 2, and 4 years following sustained virologic response (SVR) in kidney transplant recipients. Also can be found in 10.17632/n3w628yk3t.1.
