# Supplementary material for: Evaluation of Long-Term Outcomes of Direct Acting Antiviral Agents in Chronic Kidney Disease Subjects: A Single Center Cohort Study
Source: J Clin Med. 2023 May 17;12(10):3513. doi: 10.3390/jcm12103513 (PMC10219487; doi:10.3390/jcm12103513)
Supplement: Supplementary file 1 [file jcm-12-03513-s001.zip › jcm-2406396-supplementary.pdf]

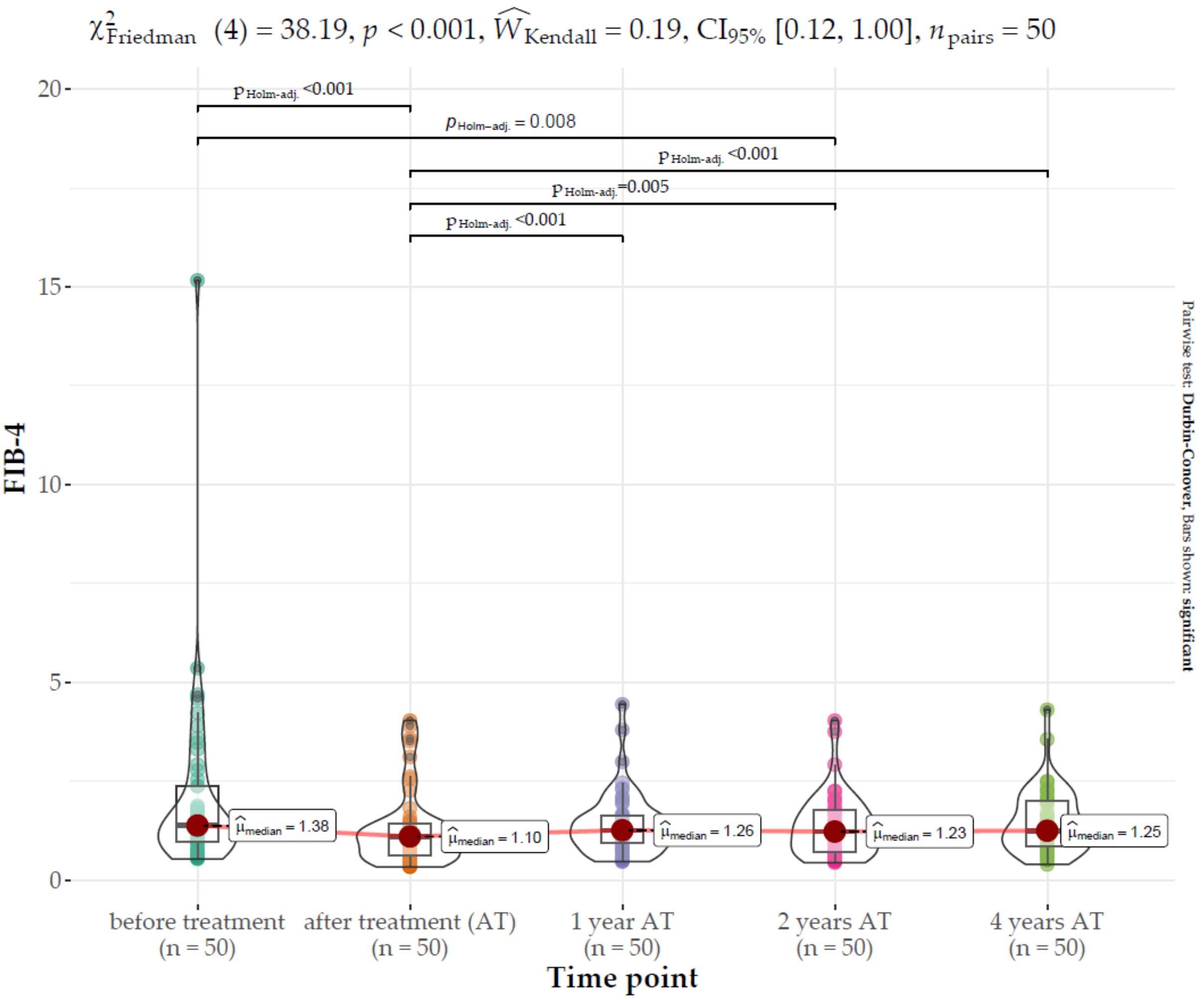

	Before treatment	After treatment	1 year AT	2 years AT	4 years AT
Mdn (IQR)	1.38 (1.42)	1.10 (0.81)	1.26 (0.71)	1.23 (1.06)	1.25 (1.16)

Figure S1. Distribution of the FIB-4 parameter at baseline, end of treatment (EOT), and 1, 2, and 4 years following sustained virologic response (SVR) in kidney transplant recipients.

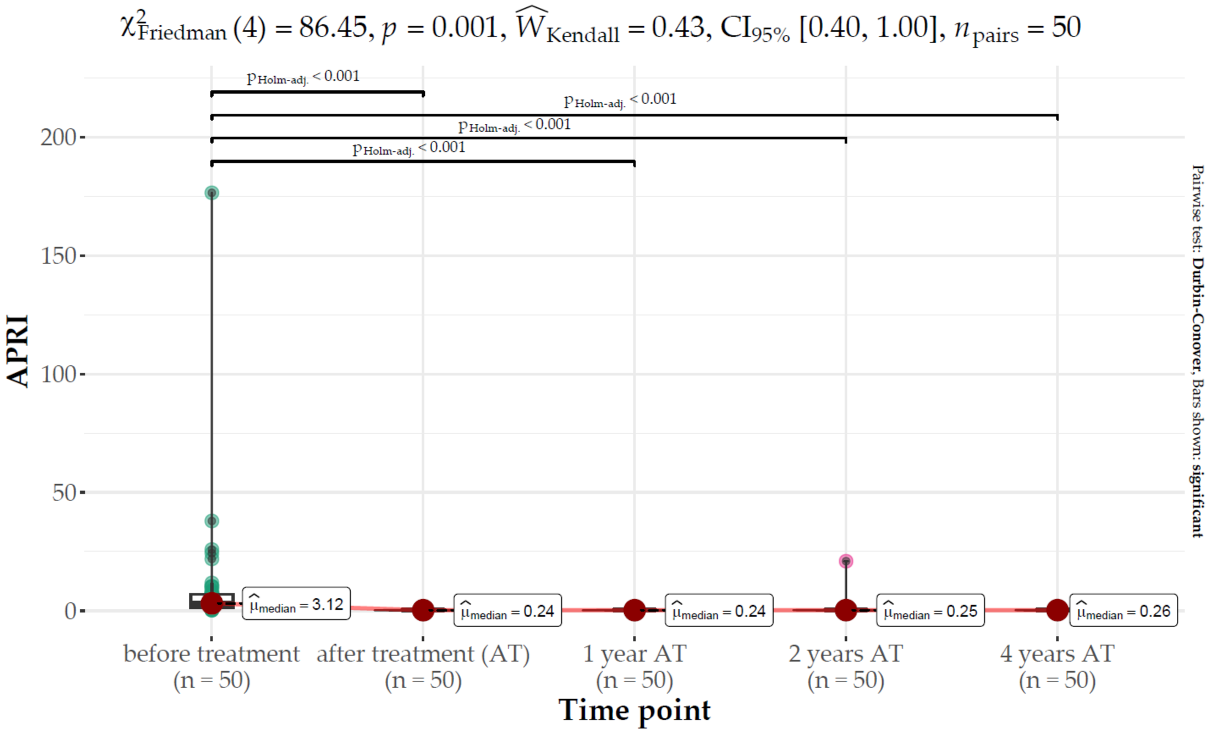

	Before treatment	After treatment	1 year AT	2 years AT	4 years AT
Mdn (IQR)	3.12 (5.57)	0.24 (0.12)	0.24 (0.13)	0.25 (0.11)	0.26 (0.13)

Figure S2. Distribution of APRI parameter at baseline, end of treatment (EOT), and 1, 2, and 4 years following sustained virologic response (SVR) in kidney transplant recipients.

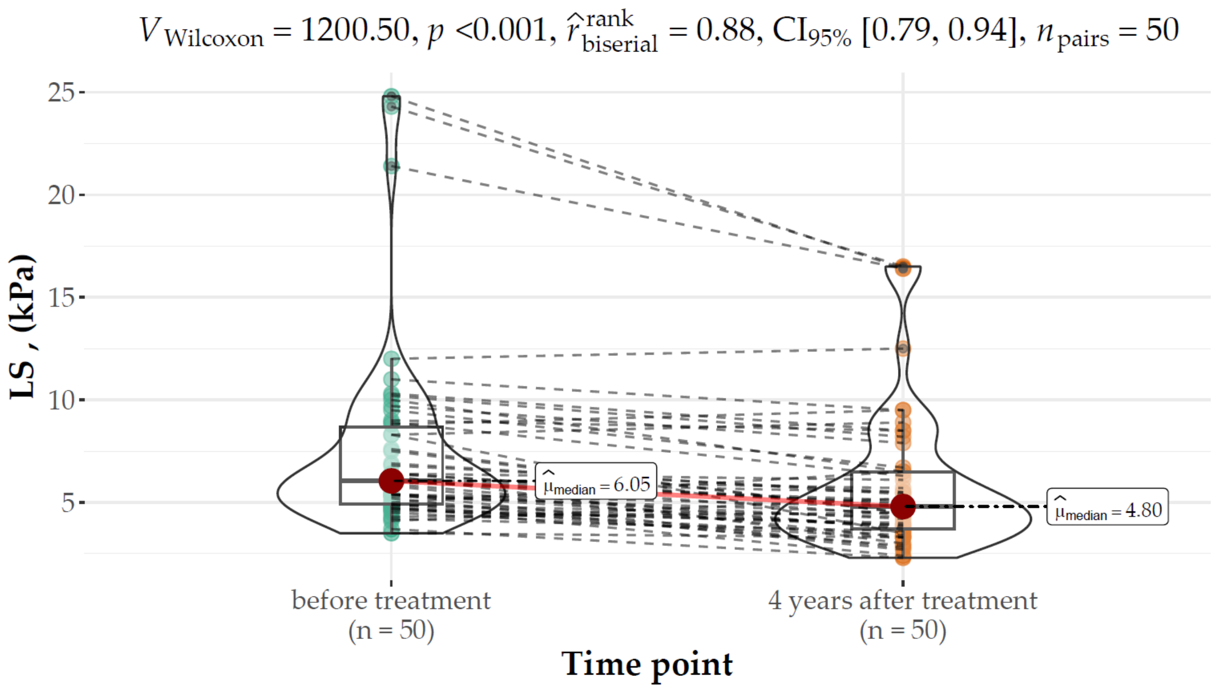

	Before treatment	After treatment
LS, Mdn (IQR); kPa	6.05 (3.75)	4.8 (2.77)

	Before treatment	After treatment
LS, Mdn (IQR); kPa	6.06 (3.75)	4.8 (2.77)

Figure S3. Distribution of LS (kPa) parameters at baseline and 4 years after the end of treatment (EOT)in kidney transplant recipients.

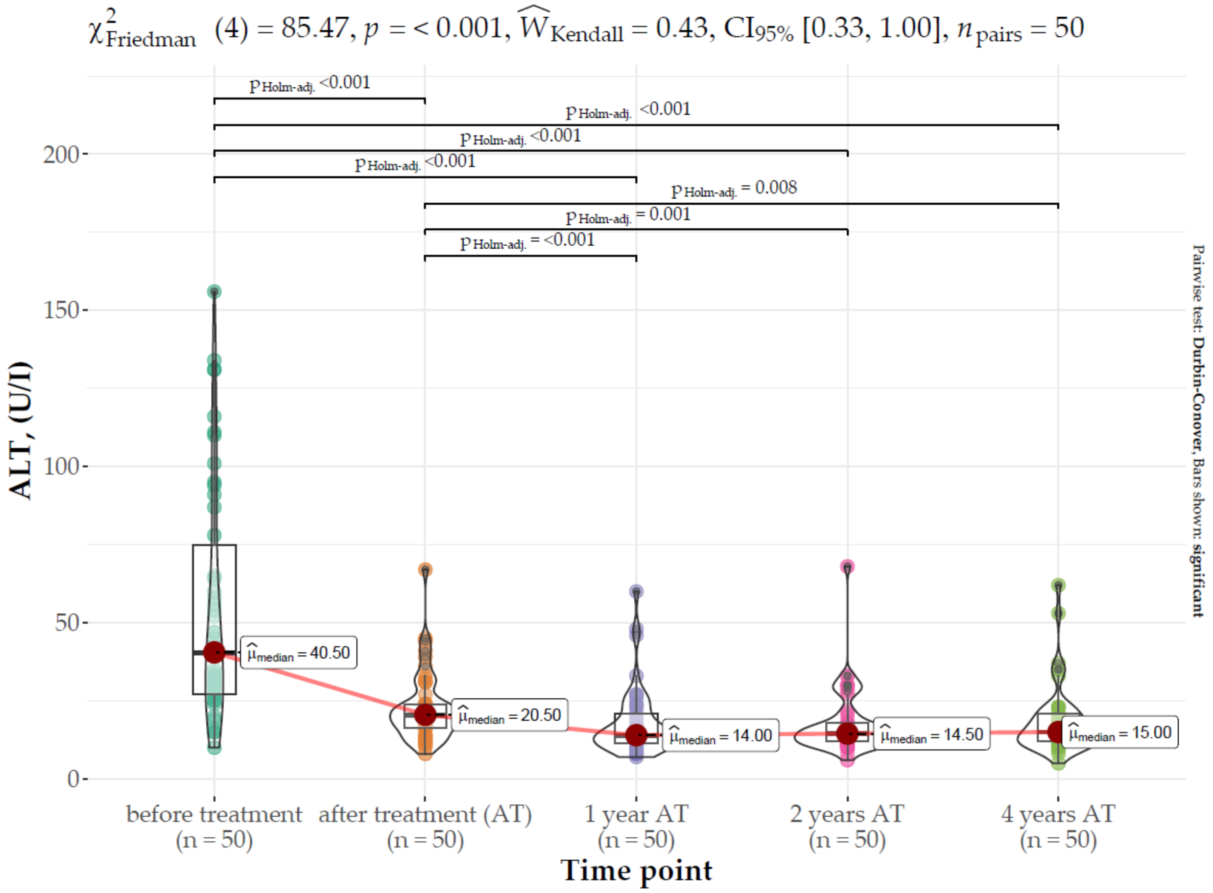

	Before treatment	After treatment	1 year AT	2 years AT	4 years AT
Mdn (IQR)	40.5 (47.75)	20.2 (0.12)	14.0 (9.50)	14.5 (6.00)	15.00 (8.75)

Figure S4 Distribution of alanine aminotransferase (ALT, U/l) at baseline, end of treatment (EOT), and 1, 2, and 4 years following sustained virologic response (SVR) in kidney transplant recipients.

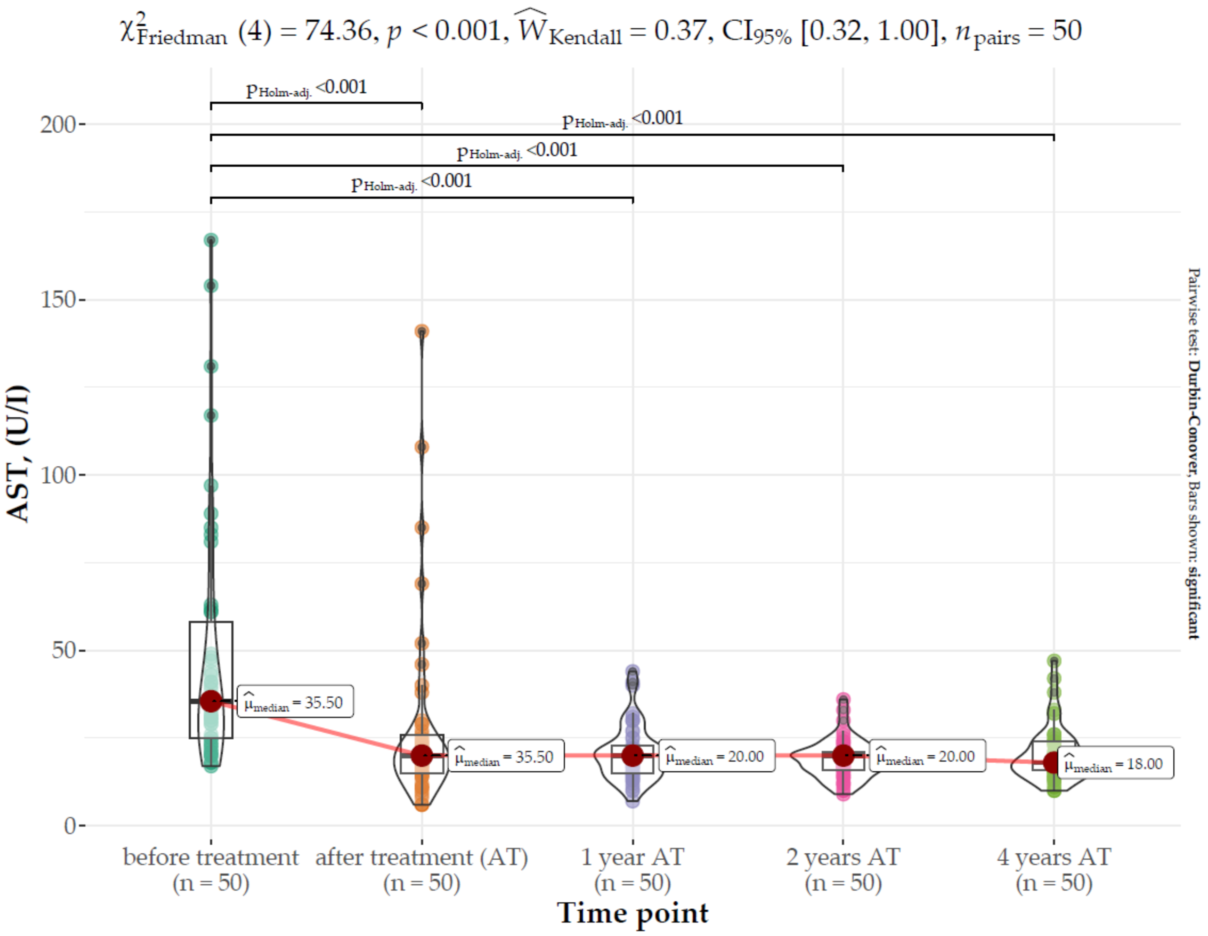

	Before treatment	After treatment	1 year AT	2 years AT	4 years AT
Mdn (IQR)	35.5 (33.7)	20.0 (10.7)	20.0 (7.7)	20.0 (5.0)	18.0 (8.0)

Figure S5 Distribution of aspartate aminotransferase (AST, U/l) at baseline, end of treatment (EOT), and 1, 2, and 4 years following sustained virologic response (SVR) in kidney transplant recipients.

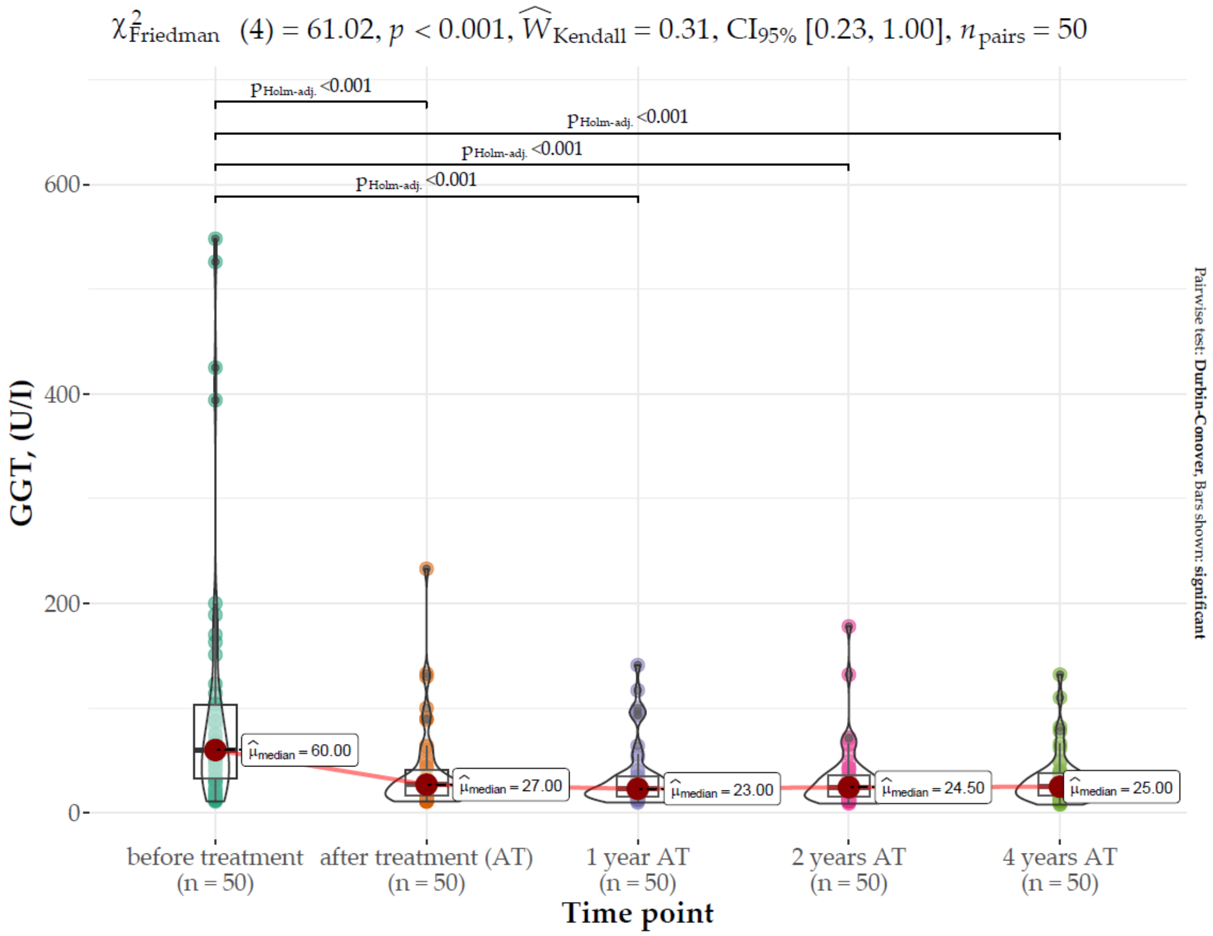

	Before treatment	After treatment	1 year AT	2 years AT	4 years AT
Mdn (IQR)	60.0 (70.5)	27.0 (24.5)	23.0 (18.7)	24.5 (20.5)	25.0 (20.5)

Figure S6 Distribution of gamma glutamyl transferase (GGT, U/l) at baseline, end of treatment (EOT), and 1, 2, and 4 years following sustained virologic response (SVR) in kidney transplant recipients.
